# Solid-State Fermentation of Wheat Bran with *Clostridium butyricum*: Impact on Microstructure, Nutrient Release, Antioxidant Capacity, and Alleviation of Ulcerative Colitis in Mice

**DOI:** 10.3390/antiox13101259

**Published:** 2024-10-17

**Authors:** Heng Zhang, Min Zhang, Xin Zheng, Xiaofang Xu, Jiawen Zheng, Yuanliang Hu, Yuxia Mei, Yangyang Liu, Yunxiang Liang

**Affiliations:** 1 State Key Laboratory of Agricultural Microbiology, College of Life Science and Technology, Huazhong Agricultural University, Wuhan 430070, China; zhang398986580@163.com (H.Z.); 13098874130@163.com (M.Z.); 15907454637@163.com (X.Z.); 15271905745@163.com (X.X.); zjiawensmile@163.com (J.Z.); mei@mail.hzau.edu.cn (Y.M.); 2 Hubei Key Laboratory of Edible Wild Plants Conservation & Utilization, College of Life Sciences, Hubei Normal University, Huangshi 435002, China; ylhu@hbnu.edu.cn; 3 Key Laboratory of Freshwater Biodiversity Conservation, Ministry of Agriculture, Yangtze River Fisheries Research Institute, Chinese Academy of Fishery Sciences, Wuhan 430223, China

**Keywords:** probiotic, phenolic compounds, ulcerative colitis, barrier function, gut microbiota, mice

## Abstract

This study investigated the effects of solid-state fermentation with *Clostridium butyricum* on the microstructure of wheat bran, the release of dietary fiber and phenolic compounds, and antioxidant capacity. Compared with unfermented wheat bran, insoluble dietary fiber and phytic acid content decreased, whereas soluble dietary fiber and water-extractable arabinoxylan content increased in *C. butyricum* culture. Because of the increased release of phenolic compounds, such as ferulic acid and apigenin, and organic acids, such as isobutyric acid, the antioxidant capacity of the culture was considerably improved. Furthermore, the culture of *C. butyricum* treated with dextran sulfate sodium-induced ulcerative colitis in mice enhanced the expression of intestinal mucus and tight-junction proteins, modulating the gut microbiota structure, increasing the levels of short-chain fatty acids in the intestine, and restoring the essential functions of the gut microbiota. These anti-inflammatory effects stemmed from the combined action of various effective components.

## 1. Introduction

Solid-state fermentation (SSF) is a bioprocess conducted in a non-liquid environment (solid matrix) using various solid materials containing carbon and nitrogen sources [1,2], such as grains, beans, fruit peels, and wheat bran, to provide the nutrients and attachment sites necessary for microorganism growth [3,4]. Compared with liquid fermentation, SSF can provide probiotics with a physical barrier and a slowly released nutritional matrix to resist adverse environmental factors, thereby enhancing the survival rate and stability of probiotics [5]. SSF offers advantages in terms of production costs, product quality, and environmental friendliness [6]. Moreover, the probiotic culture obtained through SSF comprises multiple components, including probiotics, bacterial cell debris, extracellular metabolites, and cell contents, enriched with active substances generated from the fermentation decomposition of substrates [7,8,9]. These active substances can maintain the intestinal health of the host in various aspects.

Wheat bran is a widely sourced common raw material for SSF composed of non-starch compounds, starch, proteins, and fats and is rich in trace substances such as phenolic acids, flavonoids, and phytic acid [10,11]. The active substances in wheat bran, including dietary fiber, phenolics, and flavonoids, possess antioxidant properties and intestinal protective effects, garnering significant attention in the feed and food industries [12,13]. However, the high content of crude fiber and anti-nutritive factors in wheat bran, as well as the fact that most phenolic and flavonoid active substances exist in a bound state, limits its practical application [14,15]. To release the nutrients and active substances present in wheat bran, pretreatment is often performed to enhance its value in feed or food production. One of the most common treatment methods is bioprocessing, which involves enzymatic hydrolysis and fermentation [16].

Currently, lactic acid bacteria and yeast are commonly used as probiotics for the SSF of wheat bran [2]; notably, *Clostridium butyricum*, another probiotic, can be used as a starter culture for wheat bran fermentation. With its rich enzymatic system, *C. butyricum* can produce enzymes such as amylase, protease, cellulase, and xylanase that effectively break down and release nutrients and active substances from wheat bran [17]. Simultaneously, during the SSF of wheat bran, *C. butyricum* can produce various beneficial substances, among which butyrate, its primary metabolite, is the preferred energy source for intestinal epithelial cells [18]. Butyrate has various benefits, including providing energy to cells, inhibiting the proliferation of pathogenic bacteria, improving intestinal microecological balance, and repairing damaged intestinal mucosal barriers [18]. *C. butyricum* secretes various enzymes, including cellulases, xylanases, and amylases, during the fermentation of wheat bran [19]. These enzymes break down complex carbohydrates and release fermentable monosaccharides, which support microbial growth and enhance the nutritional value of the bran. Additionally, *C. butyricum* may facilitate the release of bioactive phenols through these enzymes, imparting antioxidant and antimicrobial properties, thereby promoting the utilization of wheat bran and improving its functional characteristics [20].

This study utilized wheat bran as the raw material and subjected it to SSF using *C. butyricum* to determine its effects on the microstructure, bioactive compounds, and functional properties. Scanning electron microscopy (SEM) was employed to compare the microstructural characteristics of wheat bran before and after SSF treatment. The contents of dietary fiber, total free phenolic compounds (TPCs), and total free flavonoid compounds (TFCs) were measured. Additionally, the relative abundances of phenolic acids and flavonoids were assessed using non-targeted metabolomics. Furthermore, the changes in antioxidant and anti-inflammatory capacities were evaluated using in vitro antioxidant assays and a dextran sulfate sodium (DSS)-induced colitis model. These findings will further emphasize the application of *C. butyricum* in SSF and enhance the acceptance of *C. butyricum culture* (fermented bran, FB).

## 2. Materials and Methods

### 2.1. Chemicals and Reagents

Dextran sulfate sodium (molecular weight: 40 kDa, purity 98%) was obtained from Shanghai Yuanye Biotechnology Co. (Shanghai, China). Salazosulfapyridine (SASP) was obtained from TCI Chemicals Co. (Shanghai, China). Tumor necrosis factor alpha (TNF-α), interleukin-6 (IL-6), interleukin-10 (IL-10), inducible nitric oxide synthase (iNOS), myeloperoxidase (MPO), and enzyme-linked immunosorbent assay (ELISA) kits were obtained from Jiangsu Meimian Industrial Co. (Jiangsu, China). The RNALater Animal RNA Stabilisation Solution, RNAeasy Animal RNA Extraction Kit (centrifugal column), and 4% paraformaldehyde fixative were obtained from Beyotime Biotechnology Co. (Shanghai, China). RT-qPCR and fluorescence-based quantitative PCR kits were obtained from Vazyme Biotech Co. (Nanjing, China). Primers for qPCR were synthesized by Tsingke Biotechnology Co. (Wuhan, China). Antibodies targeting ZO-1, Claudin-1, and Muc2 were obtained from Servicebio Technology Co. (Wuhan, China). Horseradish peroxidase (HRP)-labeled goat anti-rabbit IgG was obtained from Beyotime. The other reagents were obtained from Sinopharm Chemical Reagent Co. (Shanghai, China).

### 2.2. Fermentation Procedure

*C. butyricum* was preserved in the laboratory with the preservation number CCTCC M 20241198 CB-01. The fermentation substrate comprised 50% bran, a seed age of 12 h, 3.5% maltose, 3.5% yeast extract powder, 0.2% ammonium sulfate, 0.2% calcium carbonate, and 0.2% sodium chloride. The aforementioned components were placed into a sterilization bag, mixed uniformly, and sterilized at 115 °C for 30 min before being set aside. Subsequently, the seed liquid of *C. butyricum* that was grown for 12 h was inoculated into the fermentation medium at an inoculation quantity of 3%, and the sterilization bag was placed in an anaerobic incubator and cultured at 37 °C for 48 h. Ultimately, the water content of the obtained FB was 75%. Finally, FB and unfermented bran were freeze-dried and ground for subsequent detection and use.

### 2.3. Microstructure of Bran

The microstructure of bran and FB was observed using a scanning electron microscope (SEM; JSM-6390/LV, Tokyo, Japan). The fermented sample was attached to an SEM stub, coated with gold, and placed in the SEM chamber. Photomicrographs were taken using an SEM accelerating voltage of 15 kV. The micrographs were taken at two different magnifications, 1000× and 5000×.

### 2.4. Water-Extractable Arabinoxylans, Dietary Fiber, and Phytic Acid Content Detection

Water-extractable arabinoxylans (WEAXs) were measured as described in a previous study [21]. The sample (1 mL), deionized water (1 mL), and 10 mL of the reaction solution (20% phloroglucinol ethanol solution (5 mL), chlorohydric acid (2 mL), 17.5 g/L glucose solution (1 mL), and acetic acid (110 mL)) were incubated in 100 °C for 25 min, after which the reaction was quickly terminated in an ice-water bath. The absorbance was quickly measured at 552 nm and 510 nm. Xylose was used as a standard. The content of dietary fiber was measured as described by Zhang et al., 2022 [22]. The phytic acid content was determined according to the method described by Li et al., 2024 [21].

### 2.5. Extraction of Phenolic Compounds and Content Detection

Phenolic compounds were extracted from bran according to a previously reported method with some modifications [22]. First, 20 mL of a methanol solution (80%) was added to the sample (1 g) and mixed thoroughly. The mixture was extracted using an ultrasonic extractor at 45 °C for 1 h. Then, the supernatant was collected via centrifugation (8000× *g*, 10 min), concentrated using a vacuum evaporator, and the concentrated extract was dissolved in methanol (1 mL) to obtain free phenolic compounds. Then, filter residues were added to the NaOH solution (1.5%, 10 mL), and the pH was adjusted to neutral after hydrolysis for 2 h. Finally, the supernatant was collected via centrifugation (8000× *g*, 10 min) and concentrated using a vacuum evaporator. The concentrated extract was diluted to 1 mL with ethyl acetate to obtain bound phenolic compounds. The above extracts were stored in the dark at 4 °C. TPCs were determined using the Folin–Ciocalteu colorimetric method [23]. TFCs were determined by the NaNO_2_-AlCl_3_⋅6H_2_O method [23].

### 2.6. In Vitro Antioxidant Activity Measurement

DPPH radical scavenging activity: The ability of the sample extract to scavenge DPPH radical was determined according to the reported method [24] with some modifications. Briefly, 1.0 mL of the sample was added to the same volume of an ethanolic DPPH radical solution (0.2 mM). The reaction solution was vigorously mixed and incubated at room temperature in the dark for 30 min. The control group contained an equal volume of deionized water instead of the sample. The blank group included an equal volume of ethanol instead of the DPPH radical solution. The absorbance of the solution was measured at 517 nm after centrifugation at 6000× *g* for 10 min. The scavenging ability was defined as follows:

Scavenging activity (%) = [1 − (A_sample_ − A_blank_)/A_control_] × 100

ABTS^+^ radical scavenging activity: The ABTS^+^ solution (7 mM) was mixed with potassium persulfate (2.45 mM) in an equal volume for a reaction in the dark for 12–16 h at room temperature to prepare the ABTS^+^ reagent. The ABTS^+^ reagent was diluted with ethanol to an absorbance of 0.70 ± 0.02 at 734 nm. Then, 0.1 mL of the sample extract or deionized water (as the control) was added to the ABTS^+^ reagent (1.4 mL) and mixed using a vortex mixer. After incubation at 25 °C for 30 min, absorbance was measured at 734 nm.

ABTS^+^ radical scavenging ability (%) = [(A_sample_ − A_blank_)/A_control_] × 100

### 2.7. Bacteriostatic Activity

Bacteriostatic activity was determined using the Oxford cup method. *Escherichia coli*, *Staphylococcus aureus*, *Salmonella*, *Streptococcus*, and *Vibrio* were inoculated into the LB liquid medium for 24 h. After the sterilized Oxford cup was picked up with tweezers and slightly heated near an alcohol lamp, it was placed vertically on a sterile plate so that there was no gap between it and the surface of the medium. Then, the cultured pathogen liquid was inserted into the LB medium containing 1% Agar (45 °C) at 2% inoculation amount, mixed, and poured into a sterile petri dish with an Oxford cup to cool, thus preparing the target plate. Subsequently, 200 μL of a culture supernatant (over 0.22 μm film) was added to the Oxford cup and cultured at 37 °C for 18 h. The inhibition zone was observed and measured using a vernier caliper. Three replicates were measured in each group. The pathogenic bacteria used in this study were all sourced from strains preserved in our laboratory.

### 2.8. Untargeted Metabolomics

The sample stored at −80 °C was thawed on ice. A 400 μL solution (methanol–water = 7:3, *V*/*V*) containing the internal standard was added to a 20 mg sample and vortexed for 3 min. The samples were sonicated in an ice bath for 10 min, vortexed for 1 min, and then placed at −20 °C for 30 min. The sample was then centrifuged at 12,000 rpm for 10 min (4 °C) and the sediment was removed; then, the supernatant was centrifuged at 12,000 rpm for 3 min (4 °C). A 200 μL aliquot of the supernatant was transferred for liquid chromatography–mass spectrometry (LC–MS) analysis. The model of the mass spectrometer was TripleTOF 6600+ (SCIEX, Framingham, MA, USA), and the model of the ultra-high-performance liquid chromatography (UHPLC) system was LC-30A (Shimadzu, Japan).

Data acquisition was conducted using the information-dependent acquisition mode on Analyst TF 1.7.1 Software (Sciex, Concord, ON, Canada). The original data file acquired by LC–MS was converted into mzXML format using ProteoWizard software (v3.0.8789). Peak extraction, peak alignment, and retention time correction were performed using the XCMS program (v3.18.0). The ‘SVR’ method was used to correct the peak area. Peaks with a detection rate lower than 50% in each group of samples were discarded. Subsequently, metabolic identification information was obtained by searching the laboratory’s self-built database, an integrated public database, an AI database, and metDNA. Data analysis and mapping were performed using Metware Metabolism’s cloud platform.

### 2.9. Animal Experiments

The DSS-induced UC mouse model was established as described in our previous study [25] with minor modifications. The experimental procedures were approved by the Animal Care and Use Committee of Huazhong Agricultural University (Certificate No. SYXK2020-0084) and were performed following internationally accepted guidelines and ethical principles. Sixty SPF C57BL/6 male mice (aged 6–8 weeks) were maintained in the Experimental Animal Research Centre of Huazhong Agricultural University at 25 °C under a 12 h light/12 h dark cycle, with ad lib access to standard lab food pellets [Certificate No. (2018) 06073; Beijing Keao Xieli Feed Co., Ltd., Beijing, China] and sterile water after 7 days of acclimatization. All mice were equally divided into eight groups of ten mice per group: normal group (NC); model group (MC); inactivated-culture low-dose group, including butyric acid content of 300 mg/kg; inactivated-culture high-dose group (D-H), including butyric acid content of 3000 mg/kg; *C. butyricum* group (CB-L), including *C. butyricum* content of 2.4 × 10^7^ CFU/mL; *C. butyricum* group (CB-H), including *C. butyricum* content of 2.4 × 10^8^ CFU/mL; low-dose *C. butyricum* culture group (FB-L), including butyric acid content of 300 mg/kg; and high-dose *C. butyricum* culture group (FB-H), including butyric acid content of 3000 mg/kg. To ensure that FB was not blocked by a gastric lavage needle, the culture was filtered through lavage after lyophilization. The NC and MC groups were administered 0.2 mL of 0.9% saline daily. The D and FB groups were gavaged daily with 0.2 mL of a lyophilized powder solution (1 g of lyophilized powder dissolved in 10 mL of water). The CB group was gavaged daily with 0.2 mL of the bacterial solution. For each group, body weight (BW) and food intake were recorded and fecal samples were collected daily throughout the experimental period. At the end of the experimental period, mice were fasted for 24 h, sacrificed by cervical dislocation, sterilized abdominally, and dissected, and their tissue and contents were collected and stored at −80 °C.

### 2.10. Histopathological Analysis of Colon Tissue

Colonic tissue was collected, and colon length was measured. For the intestinal inflammation assay, distal colon tissue was rinsed with 0.9% normal saline, dried on filter paper, formalin-fixed, washed under running water, ethanol-dehydrated, paraffin-embedded, cut into 5 μm slices, and stained using hematoxylin/eosin (HE). Histopathological analysis of colonic tissue samples was performed using light microscopy. Periodic acid–Schiff (PAS) staining of colonic tissue was performed by Servicebio Technology Co. (Wuhan, China).

### 2.11. Immunohistochemical Analysis

The method of Huang [26] with minor modifications was used for immunohistochemical analysis. Colon tissue sections were deparaffinized, rehydrated, treated with citrate buffer (pH 6.0) for antigen retrieval, washed with phosphate-buffered saline (PBS), incubated in 3% H_2_O_2_ to eliminate endogenous peroxidase activity, blocked with goat serum, incubated with anti-ZO-1 (catalog no. GB111402; 1:500 dilution; Servicebio) or anti-Muc2 antibody (catalog no. GB14110; 1:500 dilution; Servicebio) overnight at 4 °C, washed with PBS, covered with HRP-labeled secondary antibody, incubated at room temperature for 50 min, visualized by 3,39-diaminobenzidine (DAB) staining, counterstained with HE, and evaluated by light microscopy.

### 2.12. Enzyme-Linked Immunosorbent Assay

Blood was collected from the orbital venous plexus and centrifuged (2500× *g*, 4 °C, 10 min). The supernatant was collected and stored at −80 °C for measurements. Levels of C-reactive protein (CRP), LPS, inflammatory cytokines (TNF-α, IL-6, IL-10), and oxidative stress kinases (iNOS, MPO) were measured using ELISA kits following the manufacturer’s protocol.

### 2.13. Quantitative Real-Time PCR

Total RNA was extracted from colonic tissue using TRNzol Universal Reagent (Tiangen Biotech Co., Beijing, China) following the manufacturer’s instructions. mRNA was reverse-transcribed into cDNA using HiScript II Q RT SuperMix for the qPCR kit (Vazyme Biotech Co., Nanjing, China). PCR amplification was performed as previously described [27]. qPCR primers are described in Table 1. Relative mRNA expression was assessed using the comparative cycle method (2^−ΔΔCt^) with β-actin as the internal reference.

### 2.14. Gut Microbiota Analysis

Microbial DNA was extracted from fecal samples using the E.Z.N.A. Soil DNA Kit (Omega Bio-tek; Norcross, GA, USA). The V3–V4 regions of bacterial 16S rRNA genes were amplified using primers 338F (5′-ACTCCTACGGGAGGCAGCAG-3′) and 806R (5′-GGACTACHVGGGTWTCTAAT-3′) on a GeneAmp 9700 thermal cycler (ABI; Carlsbad, CA, USA), as described previously [27]. Purified amplicons were pooled (equimolar concentrations) and subjected to paired-end sequencing (2 × 300) on a MiSeq platform (Illumina; San Diego, CA, USA) following the standard protocols of Majorbio Bio-Pharm Technology Co. (Shanghai, China). Raw reads were deposited in the NCBI Sequence Read Archive database (accession # PRJNA1109275).

### 2.15. Levels of Short-Chain Fatty Acids in Feces

Short-chain fatty acids (SCFAs) were extracted from fecal samples and analyzed as described previously [27].

### 2.16. Statistical Analysis

This study was performed in three independent biological experiments, each consisting of three samples (*n* = 3). Data were expressed as mean ± standard deviations (SDs), and statistical analysis was performed using SPSS v.20.0 (SPSS Inc.; Chicago, IL, USA). Data were tested for normal distribution using the Shapiro–Wilk test, and the relationship between SCFA content and inflammatory cytokine levels was analyzed using the Pearson correlation test. Differences between means were considered significant for *p* < 0.05 and highly significant for *p* < 0.01. Different lowercase letters indicate significant differences (*p* < 0.05). For example, “ab” shows no significant difference with “a” or “b”, while “a” and “b” have a significant difference.

## 3. Results and Discussion

### 3.1. Changes in Structure and Nutrient Composition of Bran before and after Fermentation with C. butyricum

**Figure 1:**
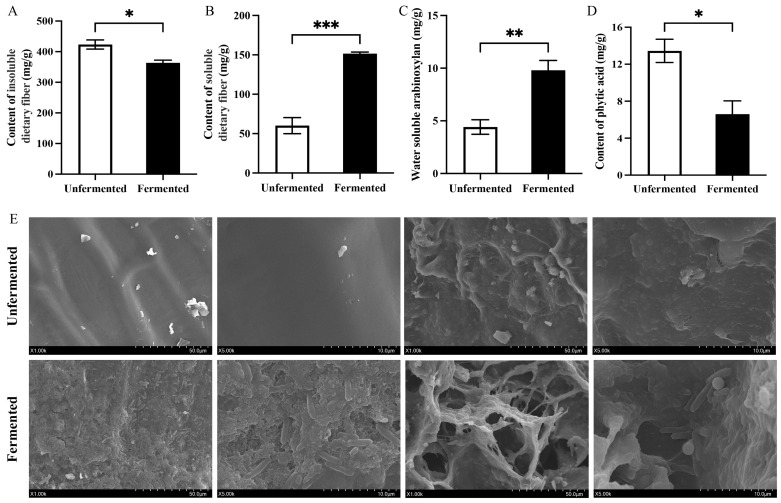
Changes in the content of IDF (**A**), SDF (**B**), WEAX (**C**), phytic acid (**D**), and microstructure (**E**) in wheat bran before and after fermentation with *C. butyricum*. IDF: insoluble dietary fiber; SDF: soluble dietary fiber; WEAX: water-extractable arabinoxylan. Values are expressed as mean ± SD. * *p* < 0.05, ** *p* < 0.01, and *** *p* < 0.001.

Grain bran contains a large amount of dietary fiber, of which insoluble dietary fiber (IDF) can increase intestinal motility, whereas soluble dietary fiber (SDF) reduces intestinal inflammation and cholesterol [28]. Figure 1A,B show SDF and IDF contents in wheat bran before and after SSF. Fermentation with *C. butyricum* significantly increased the SDF content (*p* < 0.001) from 60.22 ± 1.44 mg/g to 151.63 ± 2.70 mg/g, whereas the IDF content decreased significantly (*p* < 0.05) from 423.56 ± 4.21 mg/g to 363.72 ± 2.42 mg/g. This suggests that *C. butyricum* can produce cellulase to break down and release part of the IDF from wheat bran into SDF. It has been reported that lactic acid bacteria and yeast can also increase the SDF content in cereals by more than 30% [21]. In contrast, moderate amounts of IDF and SDF are beneficial to human health; however, excessive IDF can cause intestinal flatulence, and higher SDF levels are conducive to improving the nutrition and quality of wheat bran products [29].

Arabinoxylan, a common SDF, is the main component of wheat bran cell walls, and the oligosaccharides produced by hydrolysis are prebiotics that can selectively stimulate the growth of probiotics [30]. After fermentation with *C. butyricum*, the water-extractable arabinoxylan (WEAX) in wheat bran significantly increased (*p* < 0.01) (Figure 1C) from 4.42 ± 0.14 mg/g to 9.81 ± 0.19 mg/g. This may be due to the acidification of the fermentation mechanism (the pH of the culture is 5) and the solubilization of xylan caused by the xylanase produced by *C. butyricum* [22]. Furthermore, changes in the microstructure of wheat bran before and after fermentation also demonstrate that *C. butyricum* decomposes and uses wheat bran (Figure 1E). The SEM results showed that the structures of the smooth and rough surfaces of wheat bran were significantly disrupted, with the smooth surface becoming granular and the rough surface transforming into a porous structure. This change facilitates further fermentation by *C. butyricum* and enhances the adsorption properties and nutrient release of wheat bran [31].

In addition, the phytic acid in wheat bran is a key constituent that hinders its application, and fermentation with *C. butyricum* can significantly (*p* < 0.05) reduce the phytic acid content of wheat bran (from 13.44 ± 0.22 mg/g to 6.61 ± 0.25 mg/g) (Figure 1D). This may have occurred because *C. butyricum* can produce phytase during fermentation, and the optimal pH for phytase is 5, which is the same as the pH of the fermentation substrate. Similarly, lactic acid bacteria and yeast can produce phytase to degrade phytic acid [32,33]. In summary, *C. butyricum* can be used for wheat bran fermentation, significantly altering its structure, increasing the content of SDF and WEAX, and reducing the content of phytic acid, thereby enhancing its utilization value.

### 3.2. Evaluation of Antioxidant Activity and Bacteriostatic Capability of Fermented Bran In Vitro

**Figure 2:**
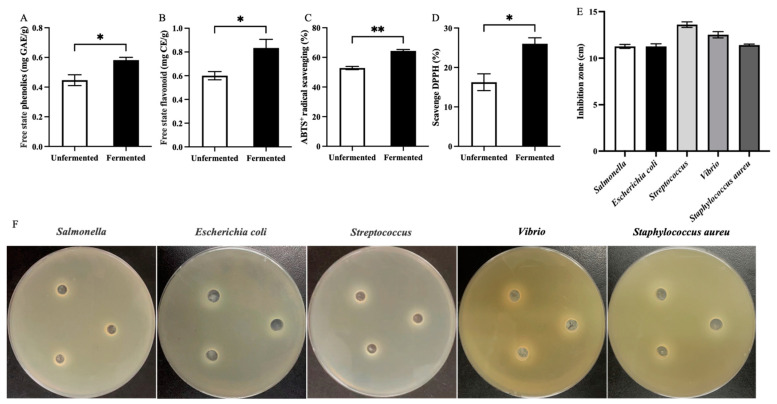
Changes in the content of TPCs (**A**) and TFCs (**B**) in wheat bran before and after fermentation with *C. butyricum* and their effects on antioxidant capacity in ABTS^+^ (**C**) and DPPH (**D**) redox models. Evaluation of the inhibitory effects of FB against pathogenic bacteria, such as *Salmonella*, *Escherichia coli*, *Streptococcus*, *Vibrio*, and *Staphylococcus aureus* (**E**,**F**). TPCs, total free state phenolics; TFCs, total free state flavonoids. Values are expressed as mean ± SD. * *p* < 0.05 and ** *p* < 0.01.

Wheat bran is rich in physiologically active phenolic compounds, including phenolic acids and flavonoids, which are usually classified into free and bound forms. Most phenolic acids and flavonoids exist as bound compounds within cellulose and can be released into free forms through enzymatic hydrolysis and fermentation [34]. Free phenolic acids and flavonoids are soluble in water or organic solvents and can be absorbed by the human body. They possess benefits, such as antioxidant properties, anti-inflammatory effects, and the prevention of fatty liver disease [35,36]. As shown in Figure 2A,B, the contents of TPCs and TFCs in wheat bran significantly (*p* < 0.05) increased after fermentation with *C. butyricum* from 0.45 ± 0.06 mg GAE/g and 0.60 ± 0.06 mg CE/g to 0.58 ± 0.03 mg GAE/g and 0.83 ± 0.13 mg CE/g, respectively. This indicates that SSF with *C. butyricum* promotes the release of phenolic acids and flavonoids from wheat bran. A similar increase in phenolic compounds has been observed in cereals fermented by lactic acid bacteria, yeast, and mold, likely due to the decomposition of the wheat bran cell wall and the hydrolysis of bound phenolic compounds during microbial metabolism, facilitating the conversion of low-molecular-weight phenolic compounds into free forms [22].

The increase in the levels of phenolic compounds suggests that FB may possess good antioxidant, antibacterial, and anti-inflammatory effects. Therefore, we evaluated the antioxidant capacities of fermented and unfermented wheat bran using DPPH and ABTS^+^ radical models. As shown in Figure 2C,D, compared with unfermented wheat bran, the scavenging ability of the FB extract against DPPH and ABTS^+^ radicals significantly (*p* < 0.01) increased from 15.62% ± 4.41% and 52.82% ± 1.88% to 25.74% ± 2.75% and 64.38% ± 1.56%, respectively, compared with the antioxidant capacity of lactic acid bacteria-fermented wheat bran reported in the literature [21,37]. These results indicate that the phenolic compounds released in wheat bran endow FB with good antioxidant ability. To evaluate the antibacterial capability of FB, we selected five pathogenic bacteria (*Salmonella*, *Escherichia coli*, *Streptococcus*, *Vibrio*, and *Staphylococcus aureus*) and performed an inhibition zone test using the supernatant of FB. The results indicated that FB had significant inhibitory effects on all bacterial species (Figure 2E,F), with inhibition zone diameters exceeding 10 cm, specifically 11.26 ± 0.37 cm, 11.27 ± 0.48 cm, 13.60 ± 0.51 cm, 12.52 ± 0.59 cm, and 11.41 ± 0.19 cm, respectively. This antibacterial ability may originate from the phenolic substances released from wheat bran or the metabolites produced by *C. butyricum*, such as ferulic acid and butyric acid. Some studies have reported that the supernatant and culture of *C. butyricum* have antibacterial effects [38]. In summary, SSF with *C. butyricum* significantly increased the release of phenolic compounds from wheat bran and enhanced the antioxidant activity and ability of FB to inhibit pathogenic bacteria.

### 3.3. Significant Increase in Phenolic Acids and Flavonoids in FB

**Figure 3:**
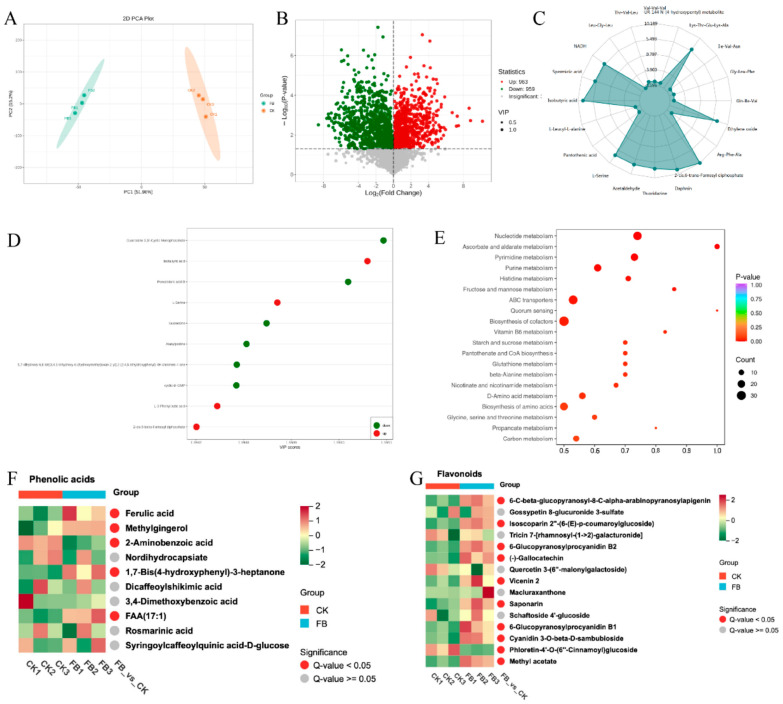
Analysis data of non-targeted metabolomics: PCA plot of metabolites before and after fermentation (**A**); volcano plot of differentially expressed metabolites (**B**); radar plot of the top 20 differentially expressed metabolites with the highest absolute log2FC values (**C**) and plot of the top 10 VIP values (**D**) in the two comparison groups; bubble plot of KEGG-based enrichment of differential metabolic pathways (**E**); correlation heatmap of the top 10 phenolic acids (**F**) and top 15 flavonoids (**G**) in terms of relative content among differentially expressed metabolites. CK, unfermented bran.

To further clarify the key metabolites increased by SSF in *C. butyricum*, non-targeted metabolomics analysis was performed on wheat bran before and after fermentation. The results revealed 5959 metabolites, including 2909 in positive ion mode and 2624 in negative ion mode. According to PCA and volcano plot analysis (Figure 3A,B), 1922 significantly different metabolites were identified, with 963 significantly increased and 959 significantly decreased metabolites in FB compared with unfermented wheat bran. This indicates that SSF with *C. butyricum* significantly alters the metabolite profile of wheat bran.

By selecting the top 20 metabolites with the highest absolute log2FC values and the top 10 metabolites with the highest VIP values in the two comparison groups for visualization (Figure 3C,D), isobutyric acid was upregulated in both comparison methods and had the highest relative content, indicating that it is an important metabolite during SSF with *C. butyricum*. Isobutyric and butyric acids are major metabolites of *C. butyricum*, belonging to SCFAs in organic acids that play a crucial role in maintaining intestinal health [18]. Furthermore, KEGG pathway annotation and enrichment analysis of differential metabolites between the two groups revealed that the top 20 pathways with the lowest *p*-values were selected for presentation. A *p*-value closer to 0 indicates more significant enrichment, and the size of the dots in the figure represents the number of differentially significant metabolites enriched in the corresponding pathways (Figure 3E). The results showed that the differential metabolites before and after fermentation were mainly enriched in pathways such as biosynthesis of cofactors, ABC transporters, nucleotide metabolism, and biosynthesis of amino acids. The enrichment of these pathways is significant for maintaining normal cellular physiological functions, promoting cell growth and proliferation, and improving metabolic efficiency.

To further explore the changes in phenolic acids and flavonoids before and after fermentation, heatmap clustering analysis was performed on the two differential metabolites. As shown in Figure 3F, the top 10 phenolic acids with the highest relative contents were selected for representation. The results obtained showed that compared with the control, the relative contents of ferulic acid, methylgingerol, 1,7-bis(4-hydroxyphenyl)-3-heptanone, and FAA in FB were significantly increased, whereas the relative content of 2-aminobenzoic acid was reduced. Among them, ferulic acid is the most abundant hydroxycinnamic acid phenolic acid in FB, originating from wheat bran and possessing good antioxidant, anti-inflammatory, and antitumor effects [39]. In addition, cluster analysis of the top 15 flavonoids with high relative contents revealed that fermentation with *C. butyricum* significantly increased the relative content of 10 flavonoid substances, including 6-C-beta-glucopyranosyl-8-C-alpha-arabinopyranosylapigenin, 6-glucopyranosylprocyanidin B2, and gallocatechin. Among them, apigenin, procyanidins, and gallocatechin have anti-inflammatory, antioxidant, and antitumor activities [40,41,42]. In summary, compared with wheat bran, the metabolites and pathways in FB are significantly altered, and phenolic compounds, such as isobutyric acid, ferulic acid, apigenin, procyanidin, and gallocatechin, are significantly increased.

### 3.4. Synergistic Action of FB Components Mitigates Colitis in Mice

**Figure 4:**
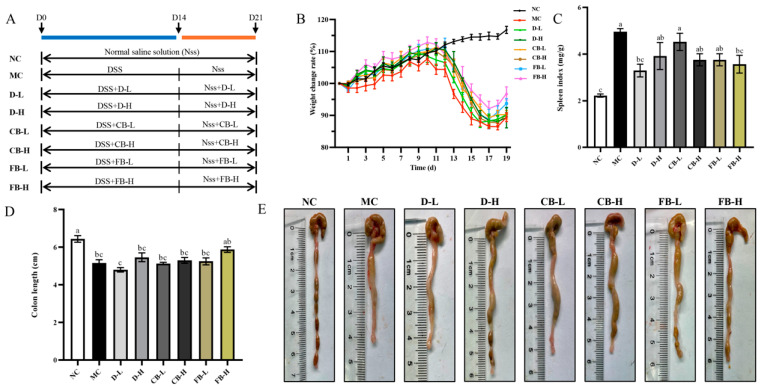
Experimental design for DSS-induced colitis in mice (**A**) and the effects of FB on body weight loss (**B**), spleen index (**C**), and colon length (**D**,**E**) in mice with colitis. Values are expressed as mean ± SD. Different lowercase letters indicate significant differences (*p* < 0.05). For example, “ab” shows no significant difference with “a” or “b”, while “a” and “b” have a significant difference.

The active ingredients in FB originate from three parts: first, the active substances released from wheat bran, such as SDF and phenolic compounds; second, the high-density *C. butyricum* and its cellular components, with the freeze-dried FB powder containing 4.81 × 10^9^ CFU/g; and third, the metabolites of *C. butyricum*, including butyric and isobutyric acids. Based on the aforementioned results, the phenolic compounds released from wheat bran and the metabolites endow FB with good antioxidant and antibacterial properties. To further investigate whether FB plays a role in alleviating intestinal inflammation and whether there is a synergistic effect between the live cells of *C. butyricum* and other components, we evaluated the live cells of *C. butyricum*, inactivated FB, and FB using a DSS-induced mouse model of ulcerative colitis. The experimental protocol is shown in Figure 4A, with each treatment group divided into high and low doses, and FB and inactivated FB quantified based on butyric acid concentration.

Since the 10th day of DSS modeling, all mice groups, except the NC group, showed a decreasing trend in BW, accompanied by symptoms, such as coarse fur, lethargy, diarrhea, bloody stool, and hunched backs, which are consistent with the typical symptoms of colitis reported in the literature [43]. As shown in Figure 4B, the BW change rate of mice in each group first increased and then decreased, and the weight loss in the MC group was consistently greater than that in the other treatment groups. These results indicate that all three intervention groups played a role in preventing weight loss in colitis mice. Among them, the weight loss rate was lower in the FB group than in the CB and D groups, suggesting that the effect of FB on reducing colitis weight loss requires the participation of live *C. butyricum* cells. Weight loss was lower in the FB-H group than in the FB-L group, indicating that metabolites, such as butyric acid, play an important role in reducing weight loss in colitis mice in a dose-dependent manner.

The organ index, which is the ratio of the weight of organs such as the liver, kidneys, and spleen to BW, along with colon length, are commonly used indicators to assess the degree of inflammatory response. Among them, the spleen, an important immune response organ, can produce antibodies and other active substances. Under normal conditions, the spleen index of mice is relatively stable, but in the presence of inflammation, the spleen may become congested, hyperplastic, enlarged, and darkened, resulting in a significant increase in the spleen index [44]. In this experiment, DSS significantly increased the spleen index (*p* < 0.01) and significantly shortened the colon length (*p* < 0.01) in mice, whereas after the intervention, colitis mice in each group recovered to varying degrees (Figure 4C–E). Among them, compared with the MC group, the spleen index of the D-L and FB-H groups was significantly reduced (*p* < 0.05) and the colon length of the FB-H group was significantly increased (*p* < 0.05).

In summary, FB had a mitigating effect on DSS-induced colitis in mice, significantly alleviating symptoms, such as weight loss, spleen enlargement, and colon shortening. This anti-inflammatory effect was derived from the synergistic action of *C. butyricum* and metabolites in the fermentation matrix.

### 3.5. FB Reduces Oxidative Stress and Inflammatory Response in Mice with Colitis

**Figure 5:**
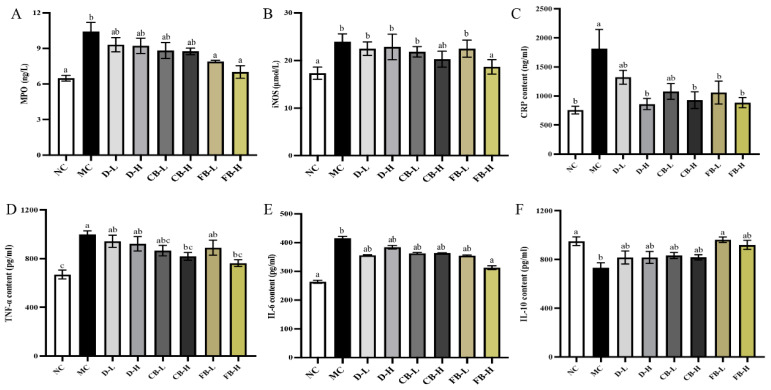
Concentrations of MPO (**A**), iNOS (**B**), CRP (**C**), TNF-α (**D**), IL-6 (**E**), and IL-10 (**F**) in the serum of mice from various groups. Values are expressed as mean ± SD. Different lowercase letters indicate significant differences (*p* < 0.05). For example, “ab” shows no significant difference with “a” or “b”, while “a” and “b” have a significant difference.

MPO and iNOS are two enzymes associated with oxidative stress reactions, and their overexpression can lead to cell and tissue damage [45]. As shown in Figure 5A,B, DSS significantly increased the levels of MPO and iNOS in mouse serum (*p* < 0.05). After treatment, the levels of MPO and iNOS decreased, with only the FB-H group showing significant reductions (*p* < 0.05). Additionally, excessive production of inflammatory factors can exacerbate inflammatory responses, leading to further colon damage [46]. The serum levels of inflammatory factors can reflect the degree of colon damage in UC mice to a certain extent. As shown in Figure 5C–E, compared with the NC group, the levels of the pro-inflammatory factors CRP, TNF-α, and IL-6 were significantly increased in the MC group, indicating that DSS administration exacerbated the inflammatory response in mice. Compared with the DSS group, the levels of CRP, TNF-α, and IL-6 were decreased in the treatment groups, but only the FB-H group showed significant reductions (*p* < 0.05). Unlike inflammatory factors, the anti-inflammatory factor IL-10 can inhibit intestinal inflammation, and a lack of IL-10 can increase susceptibility to colitis [47]. In this experiment, compared with the NC group, the level of IL-10 in the MC group was significantly decreased (*p* < 0.05), indicating that DSS exacerbated the inflammatory response by reducing the level of the anti-inflammatory factor, IL-10. After treatment in the various groups, the level of IL-10 increased, with the FB-L group showing a significant (*p* < 0.05) increase in IL-10 levels (Figure 5F). In summary, FB significantly reduced oxidative stress and inflammatory responses in mice with colitis.

### 3.6. FB Enhances Intestinal Barrier Function in Mice with Colitis

**Figure 6:**
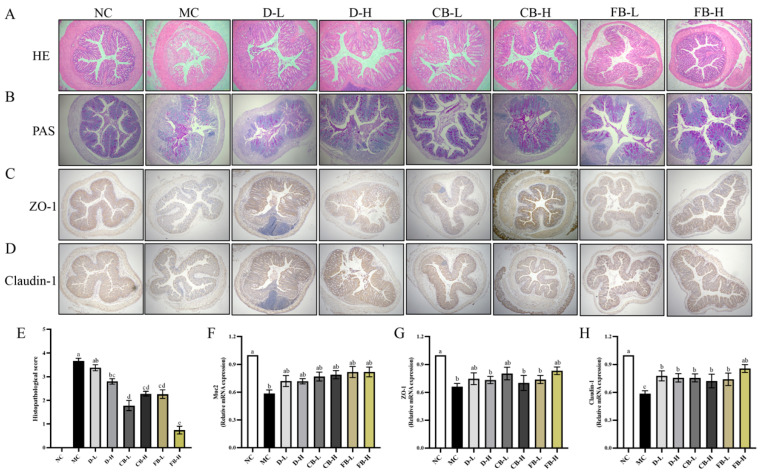
HE staining (**A**), PAS staining (**B**), immunohistochemical analysis of ZO-1 and Claudin-1 (**C**,**D**), and histological scoring (**E**) of the intestines in various groups of mice, as well as the mRNA expression levels of Muc2 (**F**), ZO-1 (**G**), and Claudin-1 (**H**). Values are expressed as mean ± SD. Different lowercase letters indicate significant differences (*p* < 0.05). For example, “ab” shows no significant difference with “a” or “b”, while “a” and “b” have a significant difference.

Pathological histological sections are commonly used to clinically assess significant tissue damage and varying degrees of cellular damage. Therefore, colon sections can be used to assess the occurrence and progression of colon cell lesions. Figure 6A shows HE-stained transverse colon sections observed under an optical microscope (×10 magnification) to determine colon structure. Mice in the NC group exhibited neatly arranged and intact colon structures, whereas the colon structure of mice in the MC group was completely destroyed, with massive inflammatory cell infiltration, intestinal mucosal injury, crypt loss, and increased histopathological scores (Figure 6E). All treatment groups showed partial damage to intestinal cells and inflammatory cell infiltration, but the colon structure of the FB-H group was more intact, with less crypt damage and fewer inflammatory cell infiltrates.

The intestinal physical barrier comprises a mucus layer and tight junctions (TJs) between intestinal epithelial cells [48]. PAS staining can observe the thickness of the mucus layer and the number of goblet cells in colon epithelial cells, whereas immunohistochemistry can detect positive regions of TJ proteins, ZO-1, and Claudin-1 in intestinal epithelial cells. The staining results showed that MC had significantly reduced mucus layer thickness and goblet cell numbers, along with similarly reduced positive regions for ZO-1 and Claudin-1 (Figure 6B–D). Compared with the MC group, the mucus layer and TJs of mice in the various intervention groups showed varying degrees of recovery, with the expression in the FB-H group being closest to that of the NC group. Similarly, the mRNA expression levels of mucin Muc2 and TJ proteins also indicated that the various intervention groups, especially FB-H, could effectively increase the expression of Muc2, ZO-1, and Claudin-1 (Figure 6F–H). Therefore, FB can effectively prevent intestinal structural damage and significantly promote mucus production and TJ protein expression, thereby enhancing intestinal barrier function.

### 3.7. FB Modulates Gut Microbiota Structure and Function in Mice with Colitis

**Figure 7:**
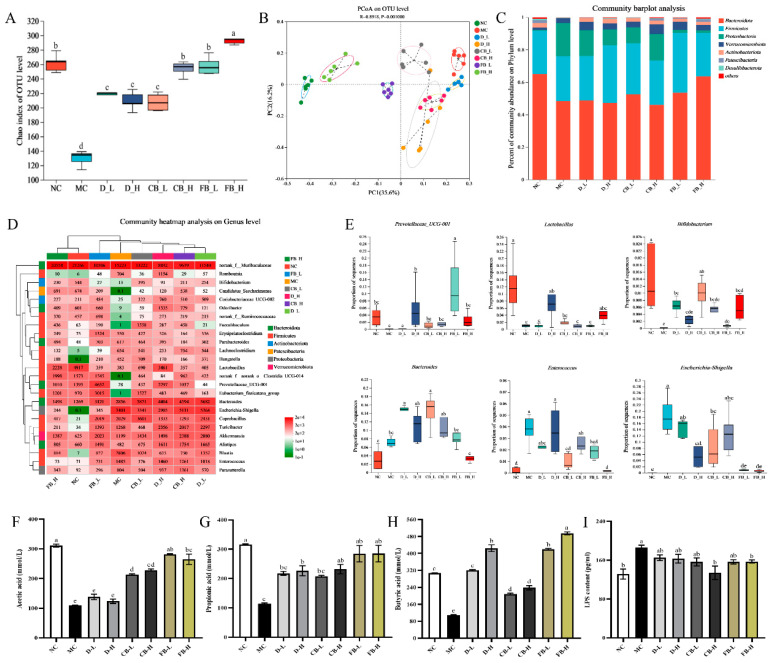
16s rRNA sequencing data revealed the Chao index (**A**) at the OTU level and the PCoA plot (**B**) of the gut microbiota in mice from various groups. In addition, a community bar plot (**C**) at the phylum level, a correlation-based clustering heatmap (**D**) at the genus level, and the relative abundance of differential bacterial genera including *Lactobacillus*, *Prevotellaceae*-UCG-001, *Bifidobacterium*, *Bacteroides*, *Escherichia-Shigella*, and *Enterococcus* were obtained (**E**). Furthermore, the concentrations of fecal microbiota metabolites, such as acetic acid (**F**), propionic acid (**G**), butyric acid (**H**), and LPS (**I**), in mice from each group were measured. The colored circles in Figure 7B are the confidence intervals of each group of samples. Values are expressed as mean ± SD. Different lowercase letters indicate significant differences (*p* < 0.05). For example, “ab” shows no significant difference with “a” or “b”, while “a” and “b” have a significant difference.

To investigate the impact of FB on the gut microbiota of mice, 16S rRNA sequencing was performed on fecal samples collected from mice after modeling. As shown in Figure 7A,B, the Chao index and PCoA were used to evaluate changes in the gut microbiota structure of mice. Compared with the NC group, the Chao index in the MC group decreased significantly (*p* < 0.01), consistent with previous reports [49]. Compared with the MC group, the Chao index in all groups increased significantly (*p* < 0.05), with the FB-H group showing a significantly higher Chao index than the other groups (*p* < 0.05). This suggests that various active components of FB can increase the α-diversity of the gut microbiota in mice with colitis and may jointly regulate the gut microbiota. PCoA is used to study the similarity or difference in community composition, where a shorter distance between two samples indicates a more similar community composition. The results obtained showed that there was a significant distance between the NC and the other groups, with the MC group being the farthest from the NC group and the FB-H group being the closest. These results indicate that the composition of the gut microbiota in the MC group underwent significant changes, and the interventions in each group could alter the gut microbiota of mice with colitis, with the combination of *C. butyricum* and matrix components in FB restoring the gut microbiota of mice with colitis toward the normal group.

Further analysis of the community composition at the phylum and genus levels was performed. At the phylum level, DSS significantly reduced the relative abundances of *Bacteroidota*, *Actinobacteriota*, and *Patescibacteria* (*p* < 0.05) and significantly increased the relative abundance of Proteobacteria (*p* < 0.01) in the mouse intestine (Figure 7C). After the intervention, the phylum-level microbiota composition of mice in all groups converged toward the NC group, with the microbiota composition of the FB-H group closest to that of the NC group. At the genus level, there were significant differences in the structure of mice in each group, with the genus-level structures of FB-H and FB-L closest to that of NC, and only FB-H exhibited the same clustering level as NC (Figure 7D). The analysis of six typical characteristic differential genera showed that the numbers of beneficial genera *Lactobacillus* (*p* < 0.01), *Prevotellaceae*-UCG-001 (*p* < 0.05), and *Bifidobacterium* (*p* < 0.01) were significantly reduced in the MC group compared with the NC group, whereas the abundance of harmful bacteria, such as *Escherichia coli*, *Shigella*, and *Enterococcus*, were significantly increased (*p* < 0.01) (Figure 7E). After intervention in other groups, these genera underwent varying degrees of change. Among them, the FB-H group significantly (*p* < 0.01) reduced the abundances of *Escherichia coli*, *Shigella*, and *Enterococcus* and increased the contents of beneficial bacteria, such as *Lactobacillus* and *Bifidobacterium*. In addition, this study further detected the contents of SCFAs (acetic, propionic, and butyric acids) and LPS in the intestinal contents of mice in each group (Figure 7F–I). The results obtained showed that except for the acetic acid content in the D-L and D-H groups, which was not significantly different from that of the MC group, all groups experienced increased contents of acetic, propionic, and butyric acids in the feces of UC mice, with the SCFAs content in FB-L and FB-H being significantly increased (*p* < 0.01). Compared with the MC group, all treatment groups reduced the LPS content in the intestinal contents of mice, but only the CB-H and FB-H groups showed significant differences (*p* < 0.05). These results indicate that FB can enrich beneficial bacteria in the gut of mice with colitis, inhibit harmful bacteria, and increase the concentration of SCFAs in the gut.

**Figure 8:**
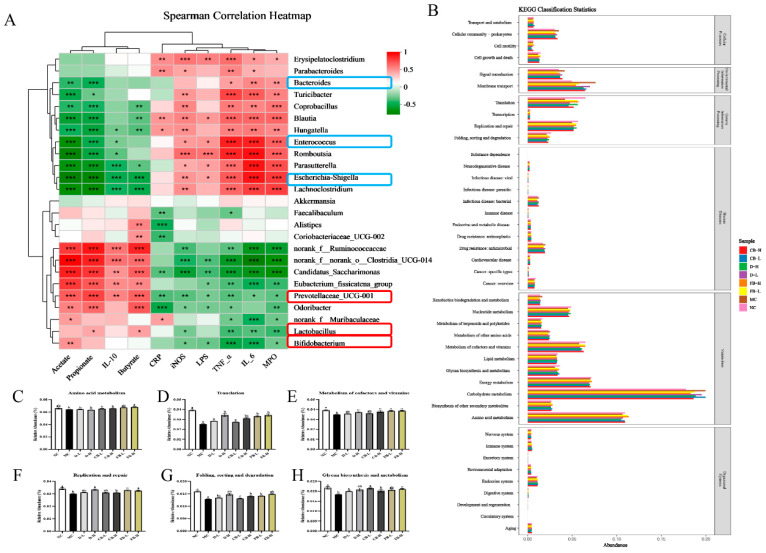
Associations between microbial genera and multiple factors are shown in a heatmap of the microbial genera (*y*-axis) that are significantly associated with different factors (*x*-axis) (**A**). The blue squares represent the genera that decreased after FB treatment in Figure 7E, and the red squares represent the genera that increased after FB treatment in Figure 7E. Level 2 KEGG orthologue functional predictions explained by PICRUSt2 (**B**). Mathematical statistics of six functional differences among the treatment groups (**C**–**H**). Values are expressed as mean ± SD. The R-value is shown in different colors in the heatmap, *p* < 0.05 is marked with *, *p* < 0.01 is marked with **, *p* < 0.001 is marked with ***, and the legend on the right is the color interval of different R values. Different lowercase letters indicate significant differences (*p* < 0.05). For example, “ab” shows no significant difference with “a” or “b”, while “a” and “b” have a significant difference.

In addition, this study employed a clustering heatmap to evaluate the correlation between the top 25 bacterial genera with the highest relative abundance and the detected indicators (Figure 8A). The results revealed a significant correlation between intestinal microbiota and the tested indicators. Bacterial genera upregulated by FB-H (*Lactobacillus*, *Bifidobacterium*, and *Prevotella*) positively correlated with SCFAs and anti-inflammatory factors, whereas downregulated genera (*Bacteroides*, *Enterococcus*, and *Escherichia coli*) positively correlated with indicators related to inflammation and oxidative stress. This demonstrates the crucial role of the gut microbiota in colitis, and the gut microbiota is a potential pivotal therapeutic target for colitis in the future [50]. Furthermore, the PICRUSt2 model was used to predict potential functional changes in the intestinal microbiota of mice in various groups. As shown in Figure 8B, based on annotations from the KEGG database, most functions belong to six primary categories: cellular processes, environmental information processing, genetic information processing, human diseases, metabolism, and organismal systems. Among the major metabolic pathways (with relative abundance greater than 3%) at the KEGG 2nd level, DSS treatment significantly reduced the relative abundance of amino acid metabolism (*p* < 0.05), translation (*p* < 0.01), cofactor and vitamin metabolism (*p* < 0.01), and replication and repair (*p* < 0.05) in the intestinal microbiota of mice. Although various treatment groups improved these four functions to varying degrees, only the FB-H intervention could significantly (*p* < 0.05) increase the relative abundance of these four functions in colitis mice (Figure 8C–F). In addition, FB-H significantly increased essential functions, such as folding, sorting, and degradation (*p* < 0.05), as well as glycan biosynthesis and metabolism (*p* < 0.05) (Figure 8G,H).

In summary, FB can regulate intestinal microbiota dysregulation by increasing microbial diversity, enriching beneficial bacteria such as *Lactobacillus*, *Bifidobacterium*, and *Prevotella*, and inhibiting harmful bacteria such as *Enterococcus* and *Escherichia coli*. This regulation improves SCFA content in the intestine, reduces LPS content, and restores essential functions such as amino acid and vitamin metabolism in mice. Compared with *C. butyricum* and inactivated FB, FB exhibited a more comprehensive modulation of the intestinal microbiota in colitis mice.

## 4. Conclusions

Wheat bran, a commonly used raw material for SSF, is an underutilized cereal by-product. SSF with *C. butyricum* is an effective method for increasing the utilization rate of wheat bran, releasing active substances, and enhancing antioxidant and antibacterial properties. Compared with wheat bran, the contents of SDF, WEAX, TPCs, and TFCs in FB were significantly increased, whereas IDF and phytic acid were partially degraded, improving the utilization value of wheat bran. SSF with *C. butyricum* enhanced the antioxidant and antibacterial activities of wheat bran by releasing phenolic compounds, such as ferulic acid and apigenin, from wheat bran, as well as producing organic acid metabolites, such as isobutyric acid. Additionally, FB derived from SSF can treat DSS-induced colitis in mice by alleviating oxidative stress and inflammatory response, enhancing intestinal barrier function, and regulating intestinal microbiota disorders to restore basic functional metabolism. Notably, the use of *C. butyricum* alone or the inactivation of FB failed to achieve the same positive regulation as FB.

## Figures and Tables

**Table 1:** qPCR primer sequences.

Target	Primer (5′—3′)	Product Length (bp)
β-Actin	F: GGCTGTATTCCCCTCCATCG	154
	R: CCAGTTGGTAACAATGCCATGT	
MUC2	F: ATGCCCACCTCCTCAAAGAC	101
	R: GTAGTTTCCGTTGGAACAGTGAA	
ZO-1	F: GAGTGGACTATCAAGTGAGCCTAA	137
	R: ATCCAAGTTGCTCGTCAATCTAA	
Claudin-1	F: CGACTCCTTGCTGAATCTGA	390
	R: CGTGGTGTTGGGTAAGAGGT	

## Data Availability

Raw reads were deposited in the NCBI Sequence Read Archive database (accession # PRJNA1109275).

## Abbreviations

FB: fermented bran (*Clostridium butyricum* culture); DSS, dextran sodium sulfate; IDF, insoluble dietary fiber; SDF, soluble dietary fiber; WEAX, water-extractable arabinoxylans; TPCs, total free state phenolics; TFCs, total free state flavonoids; MPO, myeloperoxidase; iNOS, inducible nitric oxide synthase; CRP, C-reactive protein; TNF-α, tumor necrosis factor alpha; IL-6, interleukin-6; and IL-10, interleukin-10.
